# Supplementary material for: Synthesis of Needle-like CoO Nanowires Decorated with Electrospun Carbon Nanofibers for High-Performance Flexible Supercapacitors
Source: Nanomaterials (Basel). 2024 Nov 4;14(21):1770. doi: 10.3390/nano14211770 (PMC11547584; doi:10.3390/nano14211770)
Supplement: Supplementary file 1 [file nanomaterials-14-01770-s001.zip › nanomaterials-2843948-supplementary.pdf]

Synthesis of Needle-like CoO Nanowires Decorated with Electrospun Carbon Nanofibers for High-Performance Flexible Supercapacitors

Xiang Zhang *

Department of Energy and Power Engineering, North University of China, Taiyuan 038507, China

Experimental

Materials

Polyacrylonitrile (PAN, Mw = 150,000), urea, cobalt (II) nitrate ($Co(NO_3)_2$, > 99.9%), N, and N-dimethylformamide (DMF, 99.8%) were purchased from Sigma-Aldrich and used as received. All the chemicals employed were of analytical grade and were utilized directly without further purification.

Synthesis of carbon nanofibers

The carbon nanofibers were obtained by a facile electrospinning process of PAN and subsequent annealing in Ar. Typically, 0.8 g PAN was dissolved in 10 mL DMF under stirring for 12 h. The prepared homogeneous solution was then loaded into plastic syringes (10 mL) with a 22 G needle and subsequently placed into a commercial electrospinning setup (Electrospunra, Microtools Pvt Ltd Singapore). A high-voltage power of 20 kV was applied to the needle tip. The flow rate of fluid was set to 1.0 mL h^{-1}. The humidity level inside the electrospinning chamber was 55 ± 5%. The nanofibers were collected on aluminum foil wrapped around a flat plate placed 12 cm below the needle tip. The as-electrospun PAN nanofibers were initially stabilized at 280 °C for 3 h in air. The as-stabilized sample was carbonized in a tubular furnace at 950 °C for 1 h in Ar. The heating and cooling rate was set to be 5 °C min^{-1}. After that, the as-prepared CNF was cleaned ultrasonically in 2 M HCl, washed with deionized water, and dried at 80 °C overnight.

Synthesis of CoO nanowires@electrospun carbon nanofibers

A hierarchical structure composed of cobalt oxide (CoO) nanowires embedded in electrospun carbon nanofibers (CoO NWs@CNF) was synthesized through a hydrothermal reaction followed by calcination. The schematic synthesis route is depicted in Figure 1. In this process, 2 mmol $Co(NO_3)_2{\cdot}6H_2O$, 10 mmol urea ($CO(NH_2)_2$), and 4 mmol NH_4F were added into 70 mL deionized water and stirred for 30 min at room temperature. Subsequently, the obtained homogeneous solution was transferred into a Teflon-lined stainless steel autoclave with a piece of CNF (20 mm×30 mm), immersed into the reaction solution for a hydrothermal process. The upper side of CNF was protected by a polytetrafluoroethylene tape. The Teflon-lined stainless steel autoclave was then placed in an oven at 120 °C for 8 h. After naturally cooling down to room temperature, the sample was collected via centrifugation and washed with deionized

water and alcohol to remove impurities. Finally, the sample was annealed in Ar at 350 °C for 2 h to prepare the CoO NWs@CNF.

Characterization

X-ray diffraction measurement was conducted using a Bruker AXS D8 Advance X-ray diffractometer equipped with Cu Kα radiation within the range of 20° to 80°. Raman spectra were recorded using a Dilor model OMARS 89-Z24 microprobe spectrometer, with excitation provided by an Ar^+ ion laser of 514.5 nm. The morphological features and chemical composition were examined with a field emission scanning electron microscope (FE-SEM, JEOL-6701F) using a JEOL-6701F instrument.

Electrochemical Measurements

Electrochemical measurements were conducted using a CHI 760D Electrochemical Workstation with 1 M KOH as the electrolyte. The electrospun carbon nanofiber-supported hybrid nanostructure (approximately 6 cm^2 area; CoO mass: approximately 3.0 mg cm^{-2}) directly served as the working electrode. A Pt plate and Ag/AgCl were employed as the counter electrode and the reference electrode, respectively, with all potentials referenced to the Ag/AgCl electrode. Cyclic voltammograms were recorded from -0.1 to 0.45 V at different scan rates, which ranged from 5 to 60 mV s^{-1}. Galvanostatic charge–discharge tests were performed at different current densities from -0.1 to 0.45 V. Cyclic stability was evaluated by the current density of 2 A g^{-1} for over 5000 cycles, which were acquired using an Arbin testing system (MSTAT).

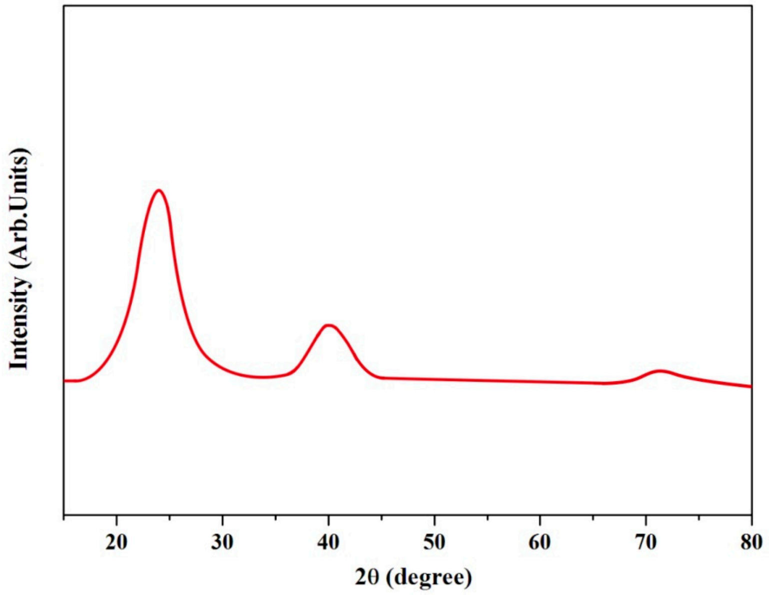

Figure S1. X-ray diffraction pattern of carbon nanofibers.

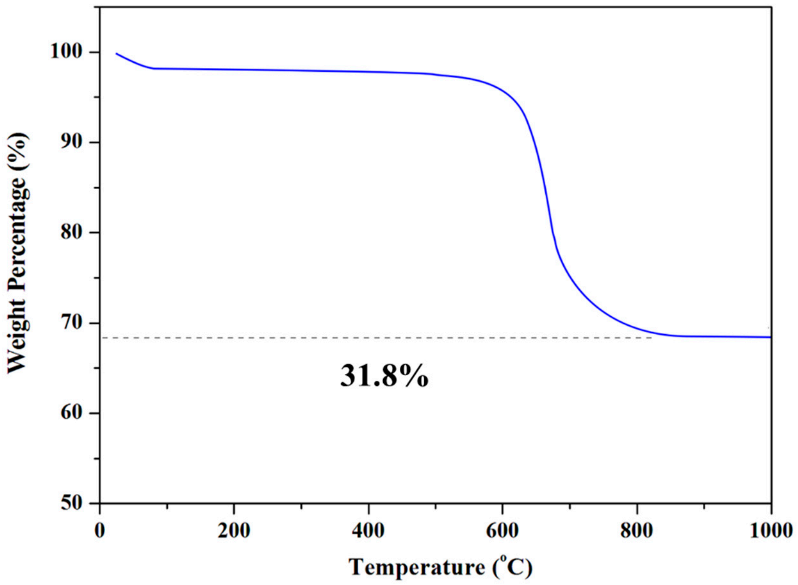

Figure S2. TGA curve of CoO NWs@CNF in air.

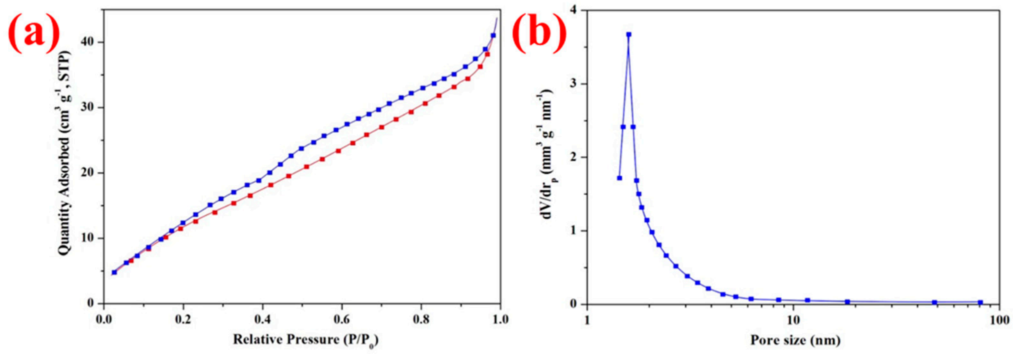

Figure S3. (a) N_2 adsorption and desorption isotherm of carbon nanofibers. (b) Pore size distribution of carbon nanofibers.

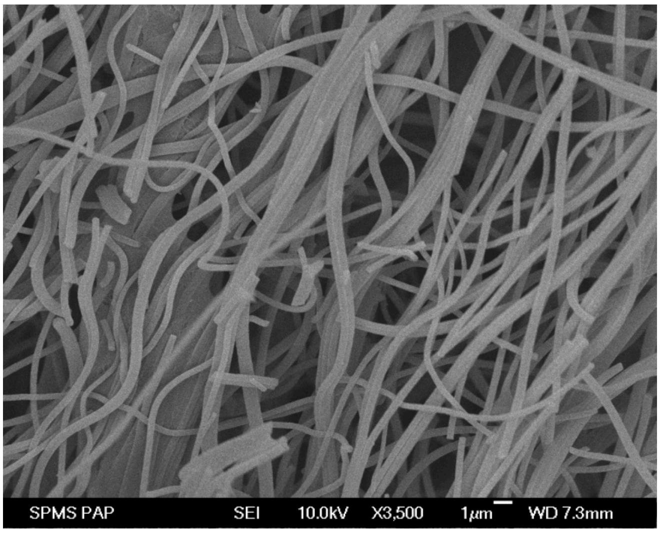

Figure S4. SEM images of as-prepared carbon nanofibers.

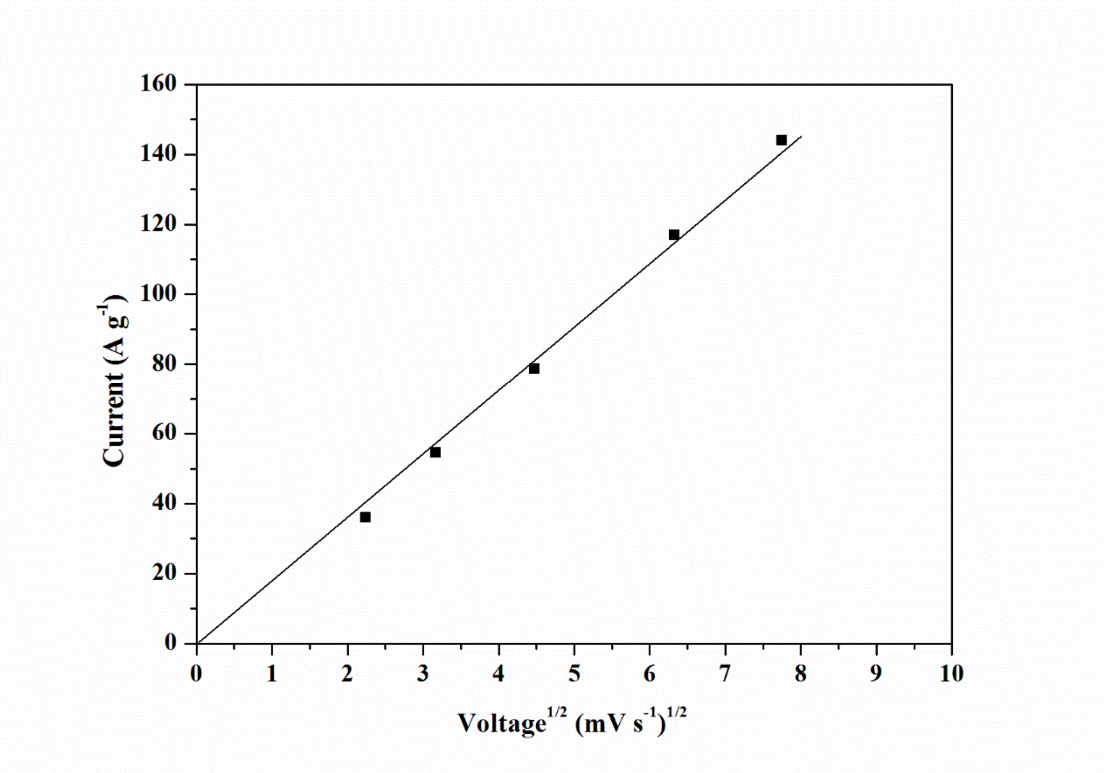

Figure S5. Dependence of the current density on voltage1/2 for CoO NWs@CNF electrode.

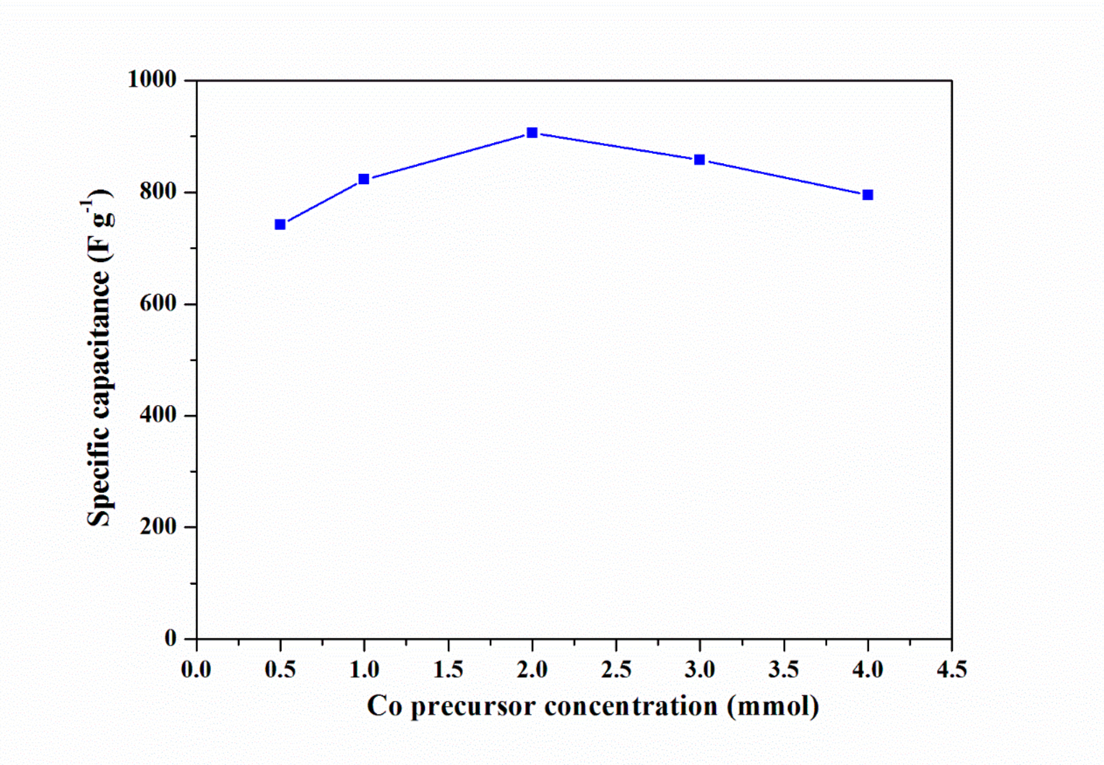

Figure S6. Specific capacitance of CoO NWs@CNF based on different precursor concentrations.

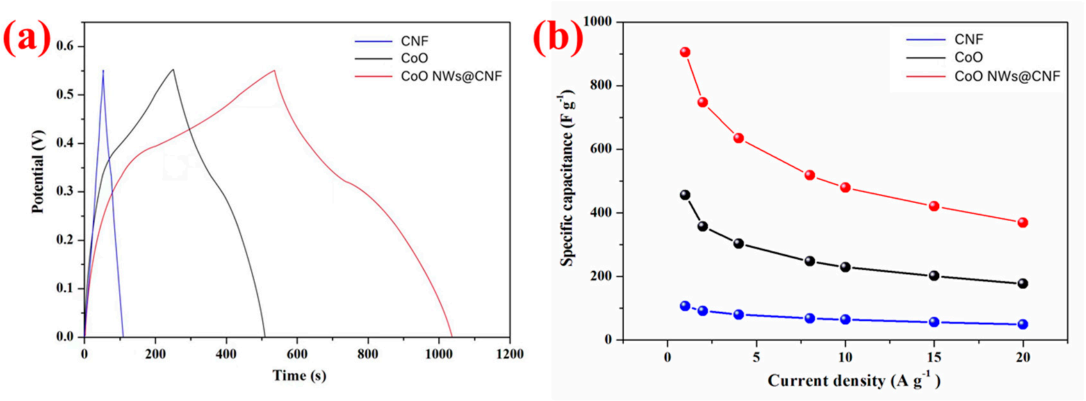

Figure S7. (a) The galvanostatic charge and discharge voltage profiles of CoO NWs@CNF, CoO nanowires, and carbon nanofibers at a current density of 1 A g^{-1}, and (b) the corresponding specific capacitance as a function of various current densities ranging from 1 A g^{-1} to 20 A g^{-1}.

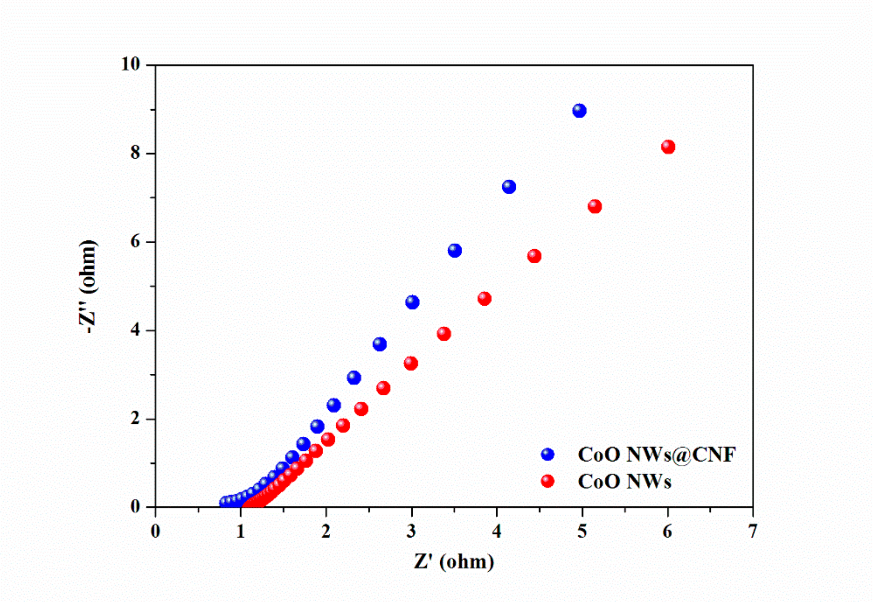

Figure S8. Nyquist plots of CoO NWs and CoO NWs@CNF

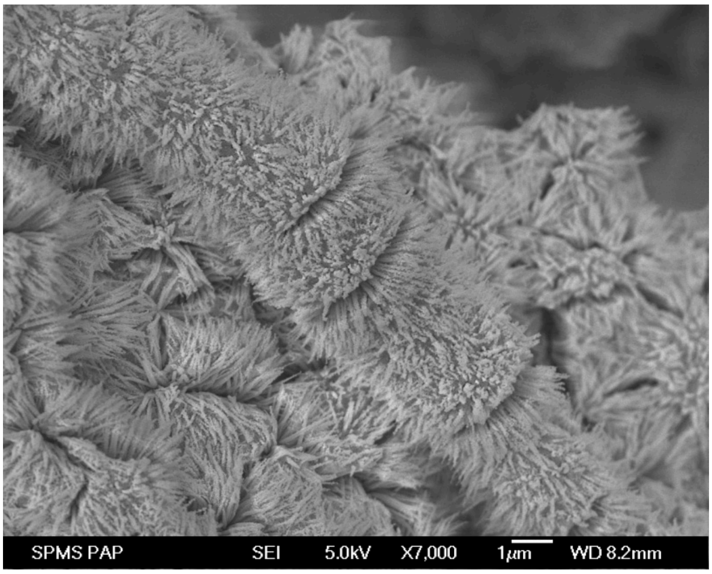

Figure S9. SEM images of CoO NWs@CNF after 10000 cycles

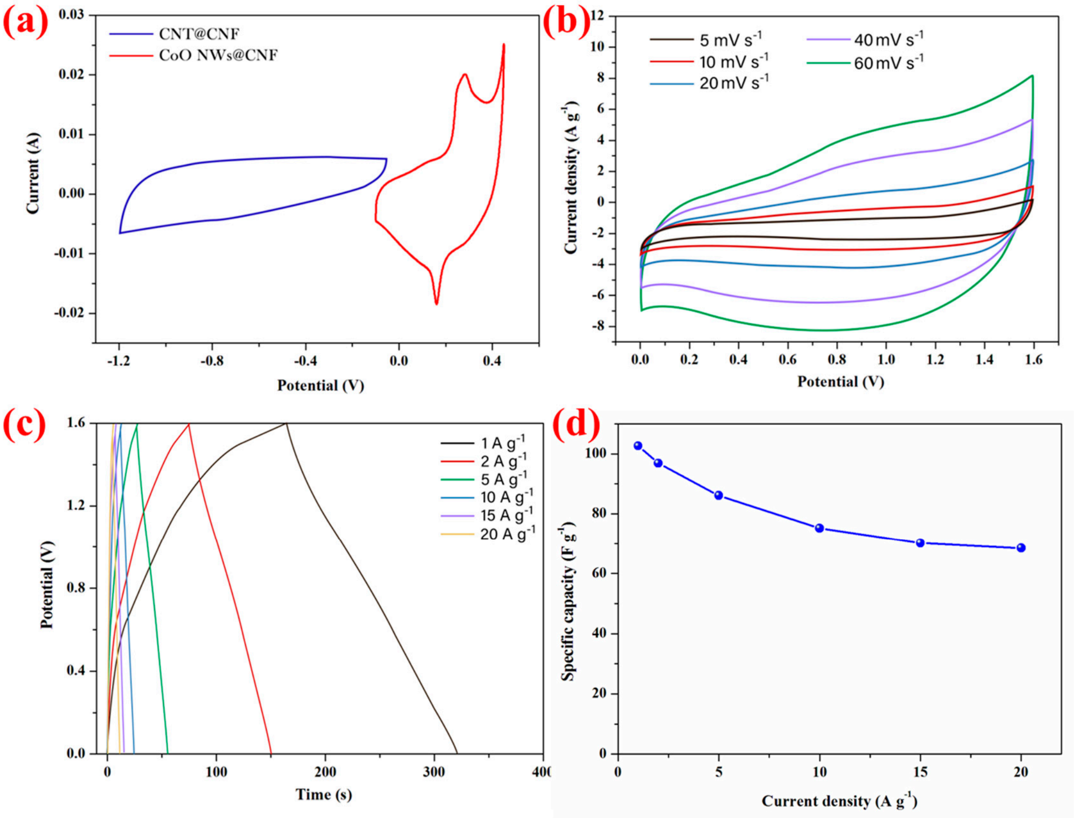

Figure S10. Electrochemical characterizations of the CoO NWs@CNF and CNT@CNF asymmetric supercapacitor. (a) Cyclic voltammetry curves of CoO NWs@CNF and CNT@CNF at a scan rate of 5 mV s^{-1}, (b) CV curves at different scan rates (5 to 60 mV s^{-1}), (c) galvanostatic charge–discharge curves at different current densities (1 to 20 A g^{-1}), and (d) specific capacitance calculated from (c) as a function of current densities ranging from 1 A g^{-1} to 20 A g^{-1}.

Table S1. Electrochemical performance of the CoO NWs@CNF in this study, compared with some other CoO electrodes reported in previous literature.

Materials	Specific Capacity (F g^{-1})	Capacity Retention after Cycling	Current density	Ref.
CoO NWs@CNF	905.8 F g^{-1} at 1 A g^{-1}	89.6 % after	2 A g^{-1}	This Study
	1068.3 F g^{-1} at 5 mV s^{-1}	5000 cycles		
	369.7 F g^{-1} at 20 A g^{-1}	~100 % after	2 A g^{-1}	
	518.1 F g^{-1} at 8 A g^{-1}	1000 cycles		
NiO@CoO on carbon cloth	912 F g^{-1} at 1 A g^{-1}<br>428.6 F g^{-1} at 20 A g^{-1}	80.9% after 5000 cycles	20 A g^{-1}	[1]
CoO nanoporous walls	539.8 F g^{-1} at 1.17 A g^{-1}<br>307 F g^{-1} at 4.55 A g^{-1}	95.7 % after 500 cycles	4.55 A g^{-1}	[2]
CoO/carbon on Ni foam	648 F g^{-1} at 0.5 A g^{-1}	100% after 1000 cycles	3 A g^{-1}	[3]
CoO nanowires@carbon cloth	311.8 F g^{-1} at 1 A g^{-1}	98.3% after 1000 cycles	6 A g^{-1}	[4]
CoO nanosheets@carbon cloth	199.8 F g^{-1} at 1 A g^{-1}	No data	No data	[4]
CoO microspheres	97.17 F g^{-1} at 1 A g^{-1}	No data	No data	[4]
Cu^0/Cu^+ co-doped CoO nanowires	695 F g^{-1} at 1 A g^{-1}	No data	No data	[5]

[1] S. Li, R. Feng, M. Li, X. Zhao, B. Zhang, Y. Liang, H. Ning, J. Wang, C. Wang, P.K. Chu, RSC Advances, 10 (2020) 37489-37499.

[2] C. Guan, X. Li, Z. Wang, X. Cao, C. Soci, H. Zhang, H.J. Fan, Advanced Materials, 24 (2012) 4186-4190.

[3] N. Zhang, X. Yan, J. Li, J. Ma, D.H.L. Ng, Electrochimica Acta, 226 (2017) 132-139.

[4] D.L. Ji, J.H. Li, L.M. Chen, D. Zhang, T. Liu, N. Zhang, R.Z. Ma, G.Z. Qiu, X.H. Liu, RSC Advances, 5 (2015) 41627-41630.

[5] W. Liu, Z. Zhang, Y. Zhang, Y. Zheng, N. Liu, J. Su, Y. Gao, Nano-Micro Letters, 13 (2021) 61.
